# Salt consumption and mortality risk in cirrhotic patients: results from a cohort study

**DOI:** 10.1017/jns.2022.69

**Published:** 2022-11-08

**Authors:** Fereshteh Pashayee-Khamene, Melika Hajimohammadebrahim-Ketabforoush, Mahdi Saber-Firoozi, Behzad Hatami, Kaveh Naseri, Sara Karimi, Saleheh Ahmadzadeh, Hamed Kord, Saeede Saadati, Azita Hekmatdoost

**Affiliations:** 1 Department of Clinical Nutrition and Dietetics, Faculty of Nutrition Sciences and Food Technology, National Nutrition and Food Technology Research Institute, Shahid Beheshti University of Medical Sciences, Tehran, Iran; 2 Digestive Disease Research Center, Digestive Disease Research Institute, Tehran University of Medical Science, Tehran, Iran; 3 Gastroenterology and Liver Diseases Research Center, Research Institute for Gastroenterology and Liver Diseases, Shahid Beheshti University of Medical Sciences, Tehran, Iran; 4 Department of Microbiology, School of Medicine, Shahid Beheshti University of Medical Sciences, Tehran, Iran

**Keywords:** Diet, Hepatic cirrhosis, Mortality, Salt

## Abstract

Since conducting a long-term randomised clinical trial is not logical and feasible to find the optimum dosage of salt intake in patients with cirrhosis, cohort studies are the best design to assess the long-term effects of dietary salt on the survival of cirrhotic patients. This cohort study aimed to evaluate the association between dietary intake of salt and mortality risk in cirrhotic patients. The present study was designed as a cohort in three referral hospitals in Iran in 2018. One hundred and twenty-one patients aged between 20 and 70 years with established cirrhosis were recruited. Dietary intakes, demographic data and disease severity were evaluated at the baseline. Participants were followed up annually. Crude survival was greater in patients with low-to-moderate salt consumption rather than in those with high consumption, and in non-consumers [34⋅26 (95 % CI 33⋅04, 35⋅49) *v*. 30⋅41 (95 % CI 27⋅13, 33⋅69) *v*. 32⋅72 (95 % CI 30⋅63, 34⋅80), *P* = 0⋅028; log-rank test]. Using the Cox proportional hazard model, it was shown that the risk of mortality in the high-salt consumption category was approximately 126 % higher than that of the reference category (non-consumers) [HR value 2⋅26, (95 % CI 0⋅91, 5⋅63)], while this risk for the low-to-moderate consumption group was about 28 % lower than the reference category [HR value 0⋅72, (95 % CI 0⋅26, 1⋅99), *P*-trend = 0⋅04]. In conclusion, a high daily dietary intake of salt might increase the rate of mortality and moderate salt restriction (instead of elimination of salt) decreases the risk of death.

## Introduction

Liver cirrhosis (LC) is a chronic disease defined with hepatocytes necrosis and fibrosis in which nutrition and dietary intakes play a pivotal role^(1,2)^. LC is a significant cause of global health burden, with more than one million deaths in 2017^(3)^. It is reported that LC is the eleventh leading cause of death worldwide^(4)^. Serum sodium concentration has been shown to be a determinant for prognosis in cirrhotic patients^(5)^. Also, sodium retention in cirrhosis is one of the most common causes of oedema and ascites^(6,7)^. Studies have shown that ascites (as a major complication in cirrhotic patients) accounts for a mortality rate of 15–20 % in 1 year to almost 44 % in 5 years since diagnosis^(8–10)^. Moreover, hyponatremia worsens the prognosis of cirrhosis so that studies have shown hyponatremic patients with LC had a mortality rate of approximately 30–45 %^(8–13)^. Although the restriction of salt and dietary sodium intake uniformly is suggested in management of ascites, the level of restriction is under debate^(14–17)^.

According to recent studies, serum sodium was found as a predicting factor of mortality and severe complications^(18,19)^. Moreover, restriction of salt intake induces less food consumption, leading to malnutrition. In addition, it has been shown that malnutrition is an independent mortality and morbidity risk factor in cirrhotic patients^(20,21)^. Thus, it seems necessary to elucidate the optimum amount of salt intake in these patients.

Since conducting a long-term randomised clinical trial is not logical and feasible to find the optimum dosage of salt intake in patients with cirrhosis, cohort studies are the best design to assess the long-term effects of dietary salt on the survival of cirrhotic patients. This cohort study aimed to evaluate the association between dietary intake of salt and mortality risk in cirrhotic patients.

## Methods

### Study population

The present study was designed as a cohort in three referral hospitals in Iran in 2018. One hundred and twenty-one patients aged between 20 and 70 years with established cirrhosis were recruited. At baseline, a general questionnaire consisting of three sections including demographic characteristics, anthropometric indices, and food frequency questionnaire (FFQ) was filled by each patient. The exclusion criteria for this analysis were pregnancy for women, suffering from cancer, pancreatitis and other chronic diseases like heart or kidney diseases for all patients. This study was conducted according to the guidelines laid down in the Declaration of Helsinki and all procedures involving human subjects/patients were approved by the Ethical Committee for Research at Shahid Beheshti University of Medical Science with the ethical code of IR.SBMU.NNFTRI.1396.186. Before enrollment, all patients signed the informed consent form.

### Dietary assessment

A reliable and valid 168-item FFQ was used for collecting the dietary intake of participants in this study^(22)^. The FFQ consisted of the food items and standard serving sizes consumed by Iranians routinely. Patients were asked to mention the frequency of consumption (daily, weekly, monthly and annually) of each food item during the previous year. Especially, the respondents were asked about the frequency of each food item that potentially enriched in salt (bread and cereals; fruits and vegetables; rice and pasta; sausages; processed meat; processed fish; cheese and other salty foods). Moreover, common salt consumption and its frequency (sodium used during cooking food or added at the table) were asked. Consumption of foods that potentially lead to salt intake was assessed in terms of frequency of intake. Daily reported portion sizes were converted into grams per day. Then, Nutrition data extracted of FFQ were analysed by using the USDA food composition table.

### Assessment of potential confounders

The severity of LC was categorised according to MELD (Model For End Stage Liver Disease) score^(2)^. Body weight was measured when the ascites fluid was tabbed. Smoking and alcohol consumption were asked because of their adverse effect on the condition of the disease.

### Follow-up and death ascertainment

In our analyses, all subjects were followed annually. Recording the vital status, patients were contacted through phone calls. Any hospital admissions and duration of hospitalisation because of complications of LC also were recorded.

### Statistical analysis

The data were analysed using the statistical package IBM SPSS, version 22.0 (Statistical Package for the Social Sciences, IBM Corp., Armonk, New York, USA). Salt consumption was reported in three categories. Since 35 % of the study population did not consume salt, it was considered as a reference category. The lowest intake was 0⋅1 g/d, and the maximum was 30 g/d with a median (Q1–Q3) of 3 (0–5), where Q1 and Q3 were the first and third quartiles, respectively. After the non-consuming group, the salt consumption from 0⋅1 to 5 and higher than 6 g were considered as low-to-moderate and high consumption categories, respectively. The Kolmogorov–Smirnov test was applied to test the normality data distribution. Differences between variables across the categories were measured if their normal distribution by the one-way ANOVA, and otherwise by the Kruskal–Wallis test. Moreover, the *χ*^2^ test was used for categorised variables. Continuous variables were reported as mean ± sd and median (Q1–Q3), and categorical ones as frequencies and percentages. Differences in survival and potential mortality rate between cirrhotic patients in the three categories were assessed by Kaplan–Meier and Cox proportional hazard model. For this analysis, the time variable was calculated as the initiate of the completion of the baseline FFQ to death incidence during the time frame of study follow-up. The event variable was the occurrence of death as well. Hazard ratios (HRs) and 95 % confidence intervals (95 % CIs) were estimated by crude, and also multivariable models that were adjusted for potential confounders, including energy, body mass index, age, the number of hospitalisations, MELD score, alcohol consumption, smoking, and gender. The proportional hazards assumption was tested using the log-rank test and a hazard plot in the Cox regression. Furthermore, in Cox regression models, instead of comparing each of the categories with the reference category, the overall trend of change across all categories (1 to 3) was tested and the *P*-trend was reported. *P*-values lower than 0⋅05 were considered significant.

## Results

**Fig. 1.**
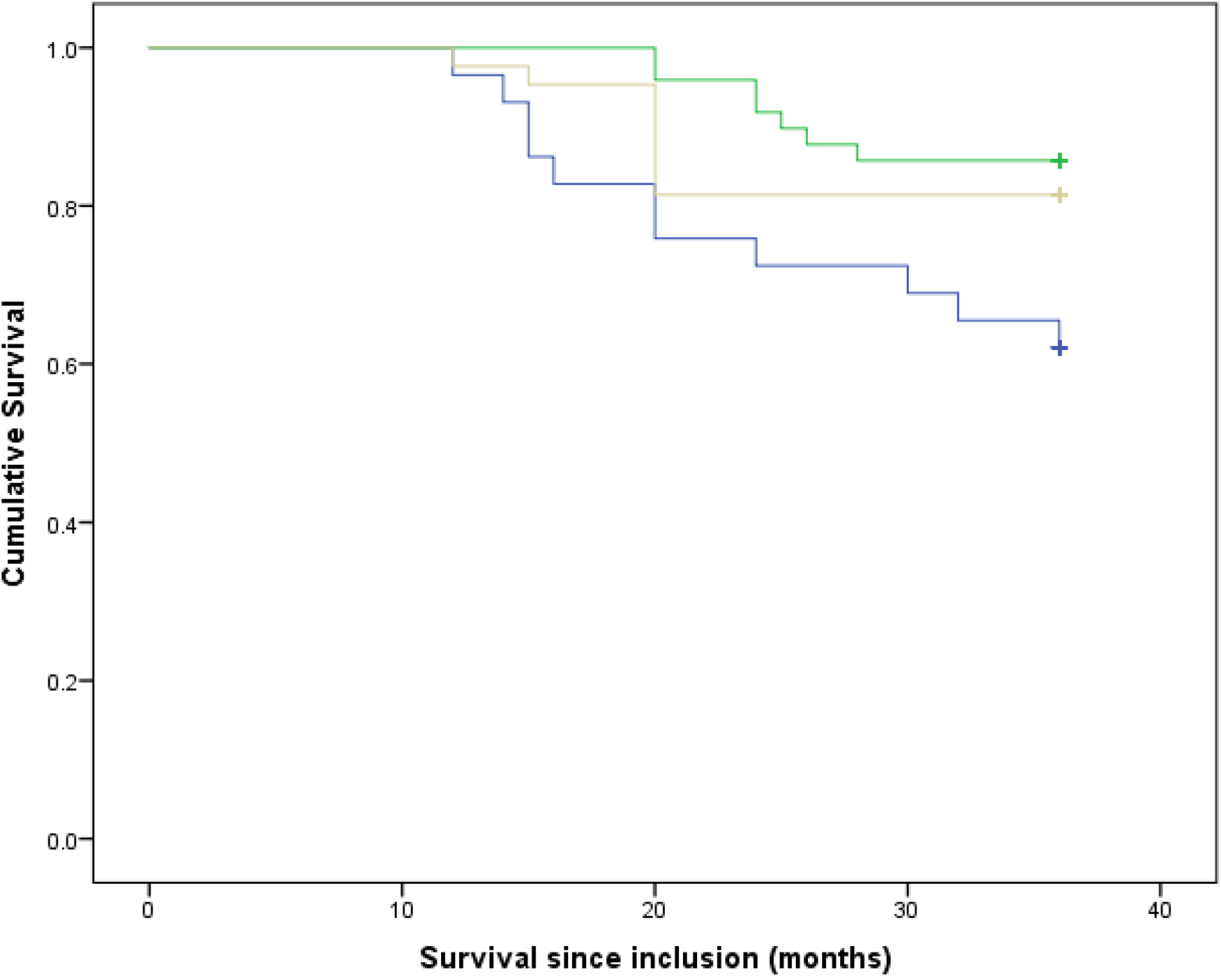
Survival rates of participants compared between different categories in terms of their salt consumption. Blue line = patients with high salt consumption; yellow line = non-consumers and green line = patients with low-to-moderate salt consumption. *P* = 0⋅028 (log-rank).

**Fig. 2.**
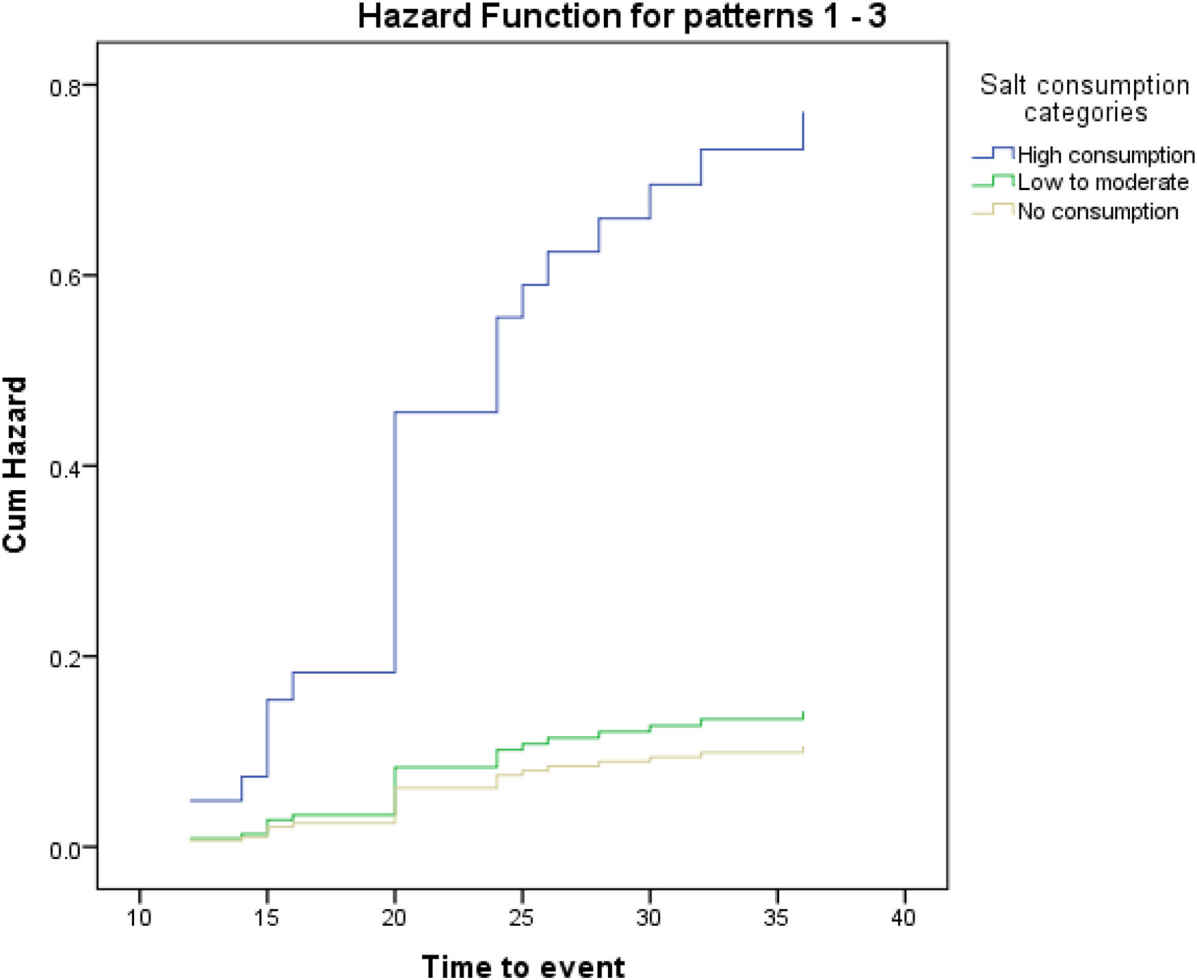
A hazard plot in the Cox regression model for salt consumption categories, by adjusted for energy, body mass index, age, number of hospitalisations, Child–Pugh score, MELD score, alcohol consumption, smoking and gender.

In the present study, 65 % of the participants reported that they consume salt. The median (Q1–Q3) salt consumption in the low-to-moderate intake group was 5 (3–5) g, and it was 10 (9⋅5–15) g in the high consumers’ quartile. Table 1 shows the basal characteristics of patients based on each category of salt consumption. Differences between the three categories were not statistically significant in terms of most of the examined characteristics; however, due to possible importance in predicting risk of death, their effect on mortality risk was considered. The median daily energy intake was highest in the high-salt consumption group. It was also higher in the low-to-moderate category than non-salt consumers, and these differences were statistically significant (*P* < 0⋅001). Also, disease severity, which was measured by MELD score, was significantly different among the three categories (*P* = 0⋅001). First of all, as shown in the Kaplan–Meier curves in Fig. 1, crude survival was greater in patients with low-to-moderate salt consumption than in those with high consumption, and even, in non-consumers [34⋅26 (95 % CI 33⋅04, 35⋅49) *v*. 30⋅41 (95 % CI 27⋅13, 33⋅69) *v*. 32⋅72 (95 % CI 30⋅63, 34⋅80), *P* = 0⋅028; log-rank test]. Thereafter, using the Cox proportional hazard model, as shown in Table 2, when the crude model was run, the results showed that the risk of mortality in the high-salt consumption category was approximately 126 % higher than that of the reference category (non-consumers) [HR value 2⋅26, (95 % CI 0⋅91, 5⋅63)], while this risk for the low-to-moderate consumption group was about 28 % lower than the reference category [HR value 0⋅72, (95 % CI 0⋅26, 1⋅99), *P*-trend = 0⋅04]. By entering energy, and then body mass index, age, number of hospitalisations, in models 1 and 2, respectively, the results were inferred approximately in much the same (Table 2). Interestingly, in the last model that was adjusted for energy, body mass index, age, number of hospitalisations, MELD score, alcohol consumption, smoking and gender, the risk of mortality in the low-to-moderate consumption category also increased compared to the reference category [HR value 1⋅35, (95 % CI 0⋅39, 4⋅70), *P*-trend = 0⋅004]. Fig. 2 shows the cumulative hazard for the three categories considering all confounders.

**Table 1.** Basal characteristics of 121 cirrhotic patients according to their salt consumption

	Salt consumption categories			*P*-value
	No consumption	Low-to-moderate consumption	High consumption	
	(*N* = 43)	(*N* = 49)	*(N* = 29)	
Age, years, mean ± sd	54⋅36 ± 12⋅03	56⋅29 ± 11⋅96	52⋅83 ± 11⋅60	0⋅44^a^
Gender, male, *n* (%)	28 (65⋅11)	33 (67⋅34)	22 (75⋅86)	0⋅61^b^
Energy intake (kcal/d), median (Q1–Q3)	1893⋅59 (1275⋅12–2213⋅34)	2425⋅71 (1974⋅71–3202⋅72)	2963⋅82 (2559⋅85–4121⋅66)	<0⋅001^c^
BMI, kg/m^2^, mean ± sd	27⋅47 ± 5⋅40	26⋅72 ± 5⋅05	27⋅40 ± 5⋅76	0⋅76^a^
Number of hospitalisations, median (Q1–Q3)	0 (0–1)	0 (0–1)	1 (0–1)	0⋅83^c^
MELD Score, mean ± sd	14⋅65 ± 5⋅88	11⋅55 ± 5⋅51	9⋅96 ± 3⋅22	0⋅001^a^
Alcohol consumption, *n* (%)	9 (20⋅93)	9 (18⋅36)	11 (37⋅90)	0⋅45^b^
Smoking, *n* (%)	13 (30⋅23)	19 (38⋅77)	15 (51⋅72)	0⋅16^b^
Ascites, yes, *n* (%)	17 (39⋅5)	16 (32⋅7)	9 (31⋅0)	0⋅70^d^

a Results from the one-way ANOVA.

b Results from the *χ*^2^ test.

c Results from the Kruskal–Wallis test.

d Results from the *χ*^2^ tests.

**Table 2.** Crude and adjusted hazard ratios of incidence death, by salt consumption (N = 121)

	Salt consumption categories			*P*-trend
	No consumption	Low-to-moderate consumption	High consumption	
	(*N* = 43)	(*N* = 49)	(*N* = 29)	
Intake, median (Q1–Q3), g	–	5⋅00 (3⋅00–5⋅00)	10⋅00 (9⋅50–15⋅00)	
Death cases, *n*	8	7	11	
HR, (95 % CI)^a^	1	0⋅72 (0⋅26–1⋅99)	2⋅26 (0⋅91–5⋅63)	0⋅04
HR, (95 % CI)^b^	1	0⋅82 (0⋅28–2⋅37)	2⋅83 (1⋅00–7⋅98)	0⋅02
HR, (95 % CI)^c^	1	0⋅81 (0⋅26–2⋅48)	3⋅03 (1⋅03–8⋅90)	0⋅01
HR, (95 % CI)^d^	1	1⋅35 (0⋅39–4⋅70)	5⋅38 (1⋅82–9⋅86)	0⋅004

a Crude model.

b Model 1: adjusted for energy.

c Model 2: adjusted for energy, body mass index, age and the number of hospitalisations.

d Model 3: adjusted for energy, body mass index, age, the number of hospitalisations, MELD score, alcohol consumption, smoking and gender. HRs (95 % CI) were calculated using Cox regression models.

## Discussion

In this prospective cohort of cirrhotic patients, the highest rate of mortality belongs to participants with the highest salt consumption. In opposite, we found the lowest rate of mortality for the low-to-moderate consumption group. To our knowledge, this is the first cohort study assessing the association between intake of dietary salt and rate of mortality in patients with LC.

Some investigations are assessing the association between serum sodium concentration and the risk of mortality. In a previous study on cirrhotic patients, serum sodium concentration was assumed as a marker for mortality risk in this population;^(23)^ however, limitation of salt intake induces less food consumption leading to malnutrition. Previous studies have shown that malnutrition is an independent risk factor for mortality in cirrhotic patients^(24,25)^. Thus, the optimum dosage of salt consumption in these patients remained to be elucidated.

In our study, we observed the highest rate of mortality in the high-salt consumption group, who consumed more than 5 g/d. On the other hand, some studies suggested that strict salt restriction might not be beneficial^(14,26)^. Severe salt restriction makes diet unpalatable and changes dietary habits and so promotes protein-calorie malnutrition and risk of mortality^(14,24,27,28)^. It has been shown that restricted sodium intake without nutritional support in comparison with unrestricted sodium intake in addition to nutritional support resulted in 3⋅9-fold higher risk of mortality within 1-year follow-up^(29)^. A review article suggested that protein and sodium have strong influences on the development of protein-calorie malnutrition, and therefore, very strict limitations of sodium should be avoided^(14)^. In our study, differences between daily energy intakes among three groups were statistically significant so that we observed the lowest daily energy intake in the non-consumer group. Some previous investigations were consistent with our findings, in which strict sodium restriction resulted in lower calorie intake^(30,31)^. In a recent study on patients with LC and ascites, a salt-restricted diet led to less calorie intake (20 %). In another study, patients with unrestricted sodium intake in comparison with the sodium-restricted group experienced more calorie intake, increased albumin levels, and shorter length of hospitalisation^(31)^.

In the last model, the non-consumer group had the lowest rate of mortality. Probably, the lack of similar results could be because of adjusting all confounders or maybe because of the low number of participants. This result may not be found if we took more patients and consequently a larger sample size in this investigation.

The present study has some strengths; the prospective nature of it, with an acceptable follow-up (about 36 months), and the vital role of sodium in changing the prognosis of the disease are the main advantages of the present study.

We have several limitations in our study. Intake of sodium was determined using FFQ, which is based on the memory of patients; however, FFQ is the most acceptable tool for assessment of dietary intake in cohort studies, the most accurate measurement of sodium intake requires collecting 24-h urine sodium excretion that was not feasible.

In conclusion, a high daily dietary intake of salt might increase the rate of mortality and moderate salt restriction (instead of elimination of salt) decreases the risk of death.
